# Horses’ (*Equus caballus*) Ability to Solve Visible but Not Invisible Displacement Tasks Is Associated With Frustration Behavior and Heart Rate

**DOI:** 10.3389/fnbeh.2021.792035

**Published:** 2021-12-08

**Authors:** Maria Vilain Rørvang, Klára Ničová, Hanna Sassner, Christian Nawroth

**Affiliations:** ^1^Department of Biosystems and Technology, Swedish University of Agricultural Sciences, Alnarp, Sweden; ^2^Department of Ethology, Institute of Animal Science, Prague, Czechia; ^3^Department of Ethology and Companion Animal Science, Czech University of Life Sciences in Prague, Prague, Czechia; ^4^Research Institute for Farm Animal Biology (FBN), Institute of Behavioural Physiology, Dummerstorf, Germany

**Keywords:** cognition, equine, heart rate, frustration, object permanence, training level, learning, equitation science

## Abstract

Many frameworks have assessed the ultimate and ontogenetic underpinnings in the development of object permanence, but less is known about whether individual characteristics, such as sex or training level, as well as proximate factors, such as arousal or emotional state, affect performance in these tasks. The current study investigated horses’ performance in visible and invisible displacement tasks and assessed whether specific ontogenetic, behavioral, and physiological factors were associated with performance. The study included 39 Icelandic horses aged 2–25 years, of varying training levels. The horses were exposed to three tasks: (a) a choice test (*n* = 37), (b) a visible displacement task (*n* = 35), and (c) an invisible displacement task (*n* = 31). 27 horses in the choice test, and 8 horses in the visible displacement task, performed significantly better than expected by chance, while none did so in the invisible displacement task. This was also reflected in their group performance, where horses performed above chance level in the choice task and the visible displacement task only. In the invisible displacement task, the group performed significantly worse than expected by chance indicating that horses persistently chose the side where they had last seen the target. None of the individual characteristics included in the study had an effect on performance. Unsuccessful horses had higher heart rate levels, and expressed more behavior indicative of frustration, likely because of their inability to solve the task. The increased frustration/arousal could lead to a negative feedback loop, which might hamper performance in subsequent trials. Care should thus be taken in future experimental designs to closely monitor the arousal level of the tested individuals in order to safeguard comparability.

## Introduction

In their daily management routines, horses face novel and sudden stimuli or objects (re-)appearing in their environment, which may elicit stress responses. Knowledge of horses’ ability to track hidden objects can thus shed light onto how horses predict the movement of visible and invisible items in different handling situations. A better understanding of how horses perceive and interpret changes in their environment could also be used to improve equine welfare and training.

Object permanence refers to the cognitive ability of mentally representing the existence and movement of hidden objects (Piaget, 1954). Such abilities enable an individual to remember the location and relocation of a resource (such as food) even when it is hidden, and therefore this ability is supposed to be highly adaptive (Rooijakkers et al., 2009). Many frameworks have assessed the ultimate and ontogenetic underpinnings in the development of object permanence, but less is known about whether individual characteristics, such as sex (Müller et al., 2011) or age, as well as proximate factors, such as arousal or emotional state (Schubiger et al., 2015), affect performance (and not necessarily comprehension) in these tasks in non-human animals. As animal cognitive research is proliferating, more and more species are added to the list of object permanence research. Many of these studies show large individual variation in performance that needs to be accounted for, e.g., which factors are associated (either in a causal or correlational way) is still sparsely investigated but is important to gather to make valid comparisons.

Object permanence was initially studied in children by developmental psychologists (Piaget, 1952). Piaget (1952) described six stages of object permanence; from 1st to 2nd stage being when a subject doesn’t search for a hidden object, to the 3rd where the subject retrieves partially hidden objects, to 4th stage when a subject is retrieving a totally hidden object, but still searching for it at previously rewarded locations—the so-called A-not-B error (De Blois et al., 1998). In the 5th stage, the subject is able to retrieve the hidden object at the correct location every time, and in the final 6th stage, the subject can mentally construct the track of a hidden object (Doré and Dumas, 1987). Stage 5 and the stages below refers to the understanding of “visible displacements” of objects, whereas stage 6 describes the understanding of “invisible displacements” of objects. The Piagetian stages are not innate but follow the development of the sensorimotor intelligence in infants (Piaget, 1954). In animals, the ability of tracking invisible displacements i.e., the 6th stage has mainly been assessed in non-human primates [chimpanzees (*Pan troglodytes*), gorillas (*Gorilla gorilla*) (Tomasello and Call, 1997), orangutans (*Pongo pygmaeus*) (De Blois et al., 1998), cotton top tamarins (*Saguinus oedipus*) (Neiworth et al., 2003)] and in a variety of bird species [pigeons (Zentall and Raley, 2019), corvids (Pollok and Gtinttirktin, 2000), psittacine birds (Pepperberg and Funk, 1990), and the Eurasian jay (Zucca et al., 2007)].

### Object Permanence in Domestic Animal Species

Object permanence has in recent years, directly or indirectly, been assessed in some domestic animal species such as dogs (*Canis familiaris*) (Triana and Pasnak, 1981; Fiset and Plourde, 2013; Zentall and Pattison, 2016), pigs (*Sus scrofa domestica*) (Nawroth et al., 2013), sheep (*Ovis orientalis aries*), and goats (*Capra aegagrus hircus*) (Nawroth et al., 2014). As these species are kept under human care, knowledge about their ability to track objects can be relevant for their welfare. Domestic dogs have been found to be able to solve visible displacement tasks where the object disappears into the container (Triana and Pasnak, 1981; Fiset and Plourde, 2013; Zentall and Pattison, 2016), and possess good memory for remembering the location of hidden and displaced object (Zentall and Pattison, 2016). Domestic pigs were tested in a set-up similar to that used for primates but adapted to the behavior of pigs (Nawroth et al., 2013). One of three locations contained a reward and pigs were tested for visible, and invisible displacement of hidden rewards, and solved only the visible displacement tasks. In goats, a later study revealed that dwarf goats performed above chance in some of the displacement experiments, where they tracked the movement of hidden objects and thus could reach stage 6 (Nawroth et al., 2015), suggesting that other domestic ungulates might possess this ability too.

In horses (*Equus caballus*), studies on object permanence are limited, although knowledge of horses’ abilities to follow hidden objects is important information for humans interacting with horses. Horses may e.g., react fearful toward re-appearing objects, conspecifics or humans, if they do not have the ability to mentally follow the objects. Such fear reactions could be unexpected by humans, which could lead to potentially dangerous situations. Uller and Lewis (2009) provided indirect evidence of object permanence, as horses in this study remembered the amount of hidden items (i.e., food). More sophisticated forms of locating objects e.g., when they are hidden and displaced, have, to the best our knowledge, only been studied by one team. This particular study showed that horses were capable of solving visible displacement of a reward on either two or three possible hiding locations but failed to solve invisible disposition tasks (Trösch et al., 2020). The results, however, need to be reviewed with caution due to the limited sample size (*n* = 16) and as only one sex was included (only females). Further investigation of object performance abilities of horses is therefore needed, and is highly relevant to the keeping and management of horses.

### What Affects Individual Performance in Cognitive Tasks?

Assessing horses’ performance in any test situation needs to be viewed in a context of individual characteristic. Individual variation means that individuals can vary in their motivation to engage in cognitive tasks, and thereby affect their performance and hence applicability (and replicability) of the results. The motivation to e.g. engage in a learning task is a crucial factor for learning as it will affect the horse’s willingness to participate, and its attention span. In a recent study by Christensen et al. (2021b), curiosity and exploration of novel objects was positively correlated with learning performance in both positive and negative reinforcement tasks. Motivation may, however, vary with both situational factors and individual characteristics such as age, sex, but also emotional state (Christensen et al., 2012a; Valenchon et al., 2013; Schubiger et al., 2015). Age has been shown to affect performance in food location tasks (Murphy et al., 2004), and in social transmission tasks (Krueger et al., 2014). Young horses also learned a practical task, e.g., the ability to use an automated forage station, quicker than older horses using the same introduction routine (Kjellberg and Morgan, 2021). In the study by Murphy et al. (2004), authors also found an effect of sex in visuo-spatial tests, with females performing consistently in six consecutive trials, whereas males performed slower in the first trial, but faster than females in subsequent trials. This effect is in accordance with findings by Wolff and Hausberger (1996) who demonstrated that females tended to learn faster than males. Collectively, this indicates that female horses are better instrumental problem solvers, while male horses outperform mares in visuo-spatial tasks. Coat colors is another individual characteristic described to be associated with greater tactile sensitivity and reactivity e.g., chestnut colored horses (Rørvang et al., 2020a). Although there is no research on the topic in horses, practitioners often report red or chestnut horses to be more reactive, and research from rodent studies show that red coat color is associated with greater pain sensitivity (Mogil et al., 2005).

In addition to the above mentioned individual characteristics, an individual’s current affective state may also affect learning performance (Schubiger et al., 2015; Ringhofer and Yamamoto, 2017). This is what has been described by the Yerkes and Dodson (1908) theory, stating that performance (in any test) increases with increasing mental arousal state, until a peak point, then decrease rapidly (i.e., the Yerkes-Dodson bell curve). Lansade and Simon (2010) found that temperament, and there the suscibility to stress, influenced learning performance but further noted that this was highly task-dependent. The latter is hypothesized to be a predisposition to react to stimuli involved in learning situations rather than a direct effect. However, it often can remain unclear which is cause and effect i.e., if stress is caused from an inability to succeed in a task, or if the stress hampers performance in the task. For example, unclear cues or unrealistic performance expectations can increase arousal level and can often result in frustration and conflict related behaviors such as kicking, pawing in ridden horses (Górecka-Bruzda et al., 2015; Christensen et al., 2021). Measuring arousal level and occurrence of frustration behavior in horses trying to solve cognitive tasks could therefore improve our understanding of how frustration can be associated with learning performance.

The current study aimed to investigate horses’ performance in visible and invisible displacement tasks and investigated whether specific ontogenetic, behavioral and physiological factors were associated with performance. The hypothesis was that horses would be able to solve visible but not invisible displacement tasks following the results from Trösch et al. (2020). In addition, the study investigated if individual characteristics such as training level, age, sex, and coat color (red or other color) influenced horses’ performance in the tasks, and if horses who were successful in one task also were successful in another. We hypothesized that training level would have a positive influence on the horse’s attentiveness toward the human and thus the task, making horses with higher training level more likely to solve the tasks. Moreover we hypothesized that younger horses would perform better than older horses, that females would perform better than males, and that red coat color would affect performance negatively in the tasks.

Lastly, the study aimed to examine if there was any association between physiological parameters (i.e., heart rate) of the horses and performance in the tests, and between behavior indicative of frustration and performance. While we did not expect any difference in horses’ heart rate between the different tests *per se*, we would expect unsuccessful horses to experience a certain level of frustration resulting in higher heart rates and more behavior linked to frustration than successful horses during the tests.

## Materials and Methods

### Ethical Considerations

Prior to the experiment start, the owner of the stud was informed and agreed to all experimental procedures, data collection and publication (written consent, signed 1st of March 2021). All procedures were conducted in accordance with national legislation on animal experimentation by the Danish Ministry of Justice, Act. no. nr. 253 (8 March 2013) and § 12 in Act. no. 1459 (17 December 2013), as well as met the ARRIVE guidelines (Kilkenny et al., 2010) and the ethical guidelines proposed by the Ethical Committee of the ISAE (International Society of Applied Ethology) (Sherwin et al., 2013).

### Corona/COVID-19 Precautions

As the experiment was conducted during the COVID-19 pandemic, measures were taken to comply with the current precautions. The experiment were conducted in Denmark, and hence complied with the Danish COVID-19 regulations effective in the experimental period 1st of March–15th of April 2021. Only one experimenter was part of the experiment to avoid contamination risks between persons. The experimenter was PCR tested for COVID-19 bi-weekly and only traveled to the stud if test results were negative (all tests = negative) before the experiment started and were never in near proximity (less than 2 m) to any other persons at the horse stud. Clothes were washed between each visit to the stud, and hands were disinfected with alcohol upon arrival and frequently during an experimental day. The experiment was conducted in a separate section of the stud, where no other daily activities connected to the stud (training and preparation for training) were performed.

### Horses and General Procedures

Thirty-nine privately owned Icelandic horses participated in the study. Thirty-two females and 7 males with a mean age of 12 years (range: 3–25 years). All horses were from the same owner and were either born at the stud where the experiment was performed, or had been trained at the stud for minimum 6 months. The stud owner was also the horses’ regular trainer. The experiment was performed in a stable section of the stud, separate from the normal daily activities going on at the stud. This stable had six individual pens with grating openings in the front of each pen toward to stable aisle. All horses were group housed when outdoors, and individually housed when indoors, in individual pens. All horses were thus acquainted with the individual pen(s) used for the experiment beforehand. All horses were also used to being alone in a pen with visual access to at least two familiar peers. Upon arrival to the stud, the owner of the horses provided data on each horse: ID, age, sex, and training level (T0–T5) (Table 1). The scale of training level was made in collaboration with the stud owner:

**TABLE 1:** Scale of training level of horses.

Training level	Description	Number of horses
Level T0	Horses were not used to any handling other than being in an indoor pen.	3
Level T1	Horses were used to being haltered and tied up in a pen.	4
Level T2	As Level 1, but in addition horses were used to wearing riding gear (harness and saddle) without a rider.	4
Level T3	As Level 2, but in addition horse were used the being ridden on a basic level (not at competition level).	16
Level T4	As Level 3, but in addition horses were used to being ridden at advanced level, and ready for competing.	8
Level T5	As Level 4; experienced in training and currently competing and signed up for evaluations.	4
**Total**		**39**

No changes were made to the horses normal feeding (roughage and concentrates were provided 6–7 am and at 5.30 pm) or training routines (normal training included either round pen training, riding (in- or outdoor) or lunging in the indoor riding arena). All horses had access to *ad libitum* water at all times.

### Principle of Experiment

**FIGURE 1:**
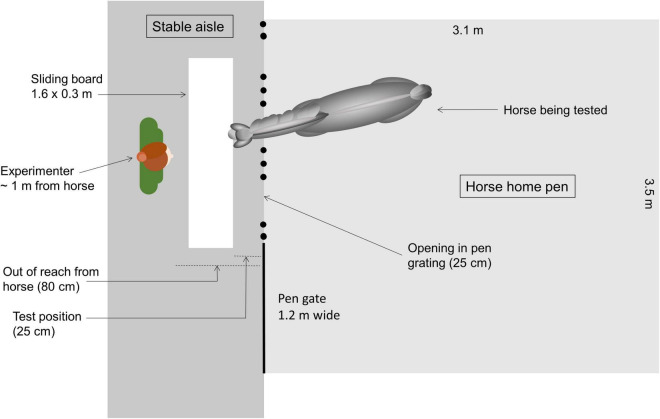
Top view of the experimental design during the tests. The horse was tested from its home pen, where the experimenter positioned herself in the stable aisle approx. 1 m away from the pen with the sliding board in between her and the horse (start position 80 cm away, test position approx. 25 cm away).

**FIGURE 2:**
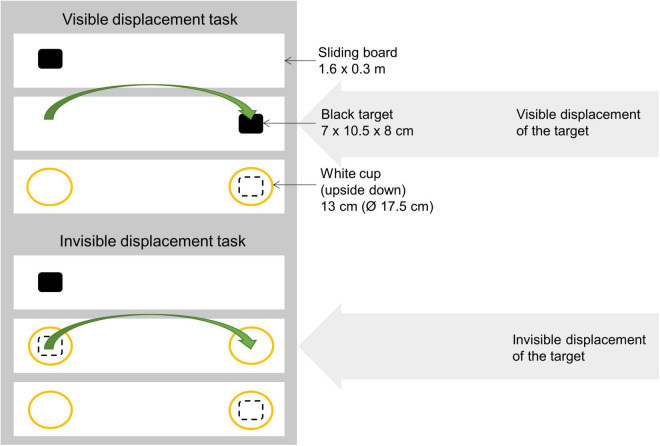
Diagram of the visible and invisible displacement tasks, illustrating how the target was moved (i.e., either visible or invisible). The sliding board, black target and white cups are illustrated, and the dashed square depicts the target while shielded. In both tasks, the last step was the experimenter pushing the board with the bowls toward the horse, allowing it to make a choice of either side of the board = one of the white cups.

The experimenter stayed on the stable aisle in front of the particular pen (Figure 1). From inside the pen, the horse had access to the aisle at three points in the pen grating (each 25 cm wide, with 70 cm between each). A white sliding board (1.6 × 0.3 m) made of lacquered wood was placed in front of the pen grating, on the aisle floor approximately 1 m away (out of reach of the horse). In the testing situation, a target (squared black plastic box: 7 × 10.5 × 8 cm, Plast team, Denmark) was placed on the board on either the left or right side. The target was then covered by a white plastic cup (height: 13 cm, Ø: 17.5 cm, Plast team, Denmark), which was also placed on the other side of the sliding board (Figure 2). The sliding board, the target and two white cups had been placed in the horse’s normal environment i.e., the stable, 48 h in advance to ensure no olfactory differences, nor olfactory cueing or novel odors. The placement of the task on floor level was chosen to accommodate the perceptual abilities of horses. Tasks where stimuli are placed at ground level appear to be better suited for the presentations to horses (Hall, 2007), probably due to the visual abilities of horses, with binocular vision only 55–65° in front of the horse (Hughes, 1977), and predominantly below the head, extending down∼75° (Timney and Macuda, 2001).

### Habituation

Horses were habituated, trained and tested in groups of 3–5 horses at a time, with at least one older and calm companion present in a nearby pen to ensure a calm atmosphere. The calm companions were always familiar horses which were physically distanced but within visual contact from the particular individual being trained or tested. The companions were either not part of the experiment (*n* = 2, who were selected by the stud owner), or horses who had already been tested to exclude any bias due to socially acquiring information about the task. Each group was habituated over a 1–2 days period, depending on progress. All horses were habituated, trained and lastly tested from their respective pen (3.1 × 3.5 m). The individual pens were bedded with wood chip bedding and had an automatic water cup adjacent to the box opening. The horse would initially be habituated to wearing a wireless heart rate monitor (Polar Equine, see section “Heart rate”) on an elastic girth, and heart rate was monitored during habituation, target training, and testing.

To prepare all horses to the procedure and the instruments used, a stepwise habituation procedure was conducted prior to the experiments:

– Step 1; In the morning, each horse was given a treat (either an apple-candy or a piece of sliced apple) placed on the aisle floor in front of a bar opening in the grating. The horse was then backed behind the grating, and given another treat on the aisle floor. This was repeated at least three, but maximum 12 times until the horse ate the treat from the aisle floor three times in a row without showing neophobic behavior (i.e., snorting, flight, freezing, backing, vigilance) (success criterion for step 1),
– step 2; the sliding board was placed in front of the grating of the pen (Figure 1), and the horse was given another treat now placed on the surface of the board or on the floor close to the board if the horse showed strong neophobic reactions. The horse was then continuously given new treats on the board, and the board was moved back and forth [i.e., in (∼25 cm from grating) and out (∼80 cm from the grating) of reach of the horse, Figure 1] between each given treat. New treats were provided until the horse complied with the final habituation criterion: promptly approaching the board, and eating the treats after these were presented, three times in a row without expressing neophobic behavior. Each horse had a maximum of 3 × 10 min to comply with the habituation criterion (mean range: 8.7, total range: 2.5–20).

### Target Training

Hereafter, a target training was initiated to teach the horse to follow a target: The horse was presented with a small black box (i.e., the target, 7 × 10.5 × 8 cm) placed in the middle of the sliding board. The target was placed on the board in full view of the horse, and the board was then pushed toward the pen grating. As soon as the horse touched the target it was rewarded with a treat (the treat was delivered by the experimenter on the board by the experimenter’s hand). The sliding board was moved away (approx. 80 cm from the grating, and out of reach of the horse, Figure 1) and the target was lifted and placed again for another trial. When the horse touched the black box within 3 s from presentation, three times in a row, the target was moved to either the right or left of the sliding board in the next trial. Hereafter, the target was presented pseudorandomly on the left or right side. When the horse touched the target 8 times in a row on either the left or right side it was considered ready to progress to testing. Each horse had two sessions of 10 trials each to reach this criterion. A video illustrating the target training can be found in Supplementary Video 1.

### Testing

#### Choice Test

Testing if the horse chooses the baited location when the target is covered/hidden: All horses received two motivation trials before the experimental session started to ensure motivation and that all horses knew that they were able to choose either of the two options (i.e., the left and the right side). In these trials, the experimenter placed the target (i.e., baited one side) in full view of the horse and pushed the board toward the horse, allowing it to make a choice. Each side was baited once in these trials without covering it, and the horse was rewarded with each correct choice. In the experimental session, the experimenter placed the target on one side of the board in full view of the horse, and covered both sides (thereby shielding the target) with white cups (each height: 13 cm, Ø: 17.5 cm), and pushed the board toward the horse (Figure 2). Each side was baited with the target pseudorandomly five times per session (with the limitation of baiting the same side 2 times in a row), after which there was a 5 min break. The horse made its choice by placing its muzzle/head over or on the white cup and obtained the reward only if it chose correctly. In order to be successful, horses needed to achieve at least eight out of 10 correct choices in two consecutive sessions. Each horse had a maximum of 4 sessions to be successful (= 20 trials). The first 10 trials for each horse was used in the subsequent analyses.

#### Visible Displacement Task

Testing if the horse follows the target when it changes position: The procedure followed that of the choice test (above), but in this case, the target and the experimenter’s one hand was simultaneously placed on both sides of the board (Figure 2), thereby interacting with both sides of the board equally often. Hereafter, the target and the hand changed position on the board in full view of the horse (left to right or right to left). The experimenter used the ipsilateral hand to place the target, and performed the first half of the displacement with the ipsilateral hand, and then the contralateral hand to avoid cueing for particular hands. The target was then covered (Figure 2) and the board was moved toward the horse, allowing it to make a choice. The horse made its choice by placing its muzzle over or on a white cup and obtained the reward only if it chose correctly. This trial was repeated 10 times with reward position and cross direction (toward or away) randomized for each horse across age, sex, and training level. All other procedures were similar to the ones described in the choice test.

A video illustrating a successful completion of a trial in the visible displacement task can be found in Supplementary Video 2.

#### Invisible Displacement Task

Testing if the horse follows a hidden target when it changes position. In this last experiment, the horses were presented with an invisible displacement, where two identical cups crossed paths on the sliding board. The target was placed in either the left or the right side of the board while the other side was also touched by the experimenter’s hand as in the visible displacement task (Figure 2). Both sides were then covered by the white cups simultaneously, and secondly the experimenter moved the cups simultaneously to the left/the right side, respectively, resulting in the cups crossing paths on the middle of the board. The experimenter again used the ipsilateral hand to place the target, and performed the first half of the displacement with the ipsilateral hand, and then the contralateral hand to avoid cueing for particular hands. Two seconds after the invisible displacement, the experimenter pushed the board toward the horse allowing to make a choice. This trial was repeated 10 times (as in the visible displacement task) with reward position and cross direction (toward or away) randomized for each horse across age, sex, and training level. All other procedures were similar to the ones described in the choice test.

A video illustrating a successful completion of a trial in the invisible displacement task can be found in Supplementary Video 3.

### Behavioral Observations

Video recording of all habituation, training and tests was conducted from the stable aisle, using a Sony HDR-cx240E handycam on a tripod (150 cm high). After each experimental day, video files were downloaded and raw data was extracted into Excel (version 2016) by an experienced observer. Trials needed to reach the step 1 habituation criterion were counted, and the duration of time needed for the horse to accomplish the step 2 habituation criterion was measured. During target training, horses had different ways of approaching the task. Some were more active and had more trials per minute than others had; hence, trials per minute were noted for each horse to form a variable indicative of the individual’s motivation to the task (mean trials per minute during target training). Success rate in target training was also noted as successful trials out of 10 trials total (as all horses accomplished at least 80% success rate in their first session i.e., 10 trials). For all tests; choice test, and visible and invisible displacement task, success rate (successful trials out of 10) was noted, and behavior potentially indicative of frustration (defined as; scraping or stamping the pen bedding, scratching body, head shaking, or pawing (from Rørvang et al., 2020a,b) was also noted as 1/0 sampling during each trial (hence maximum 10 occurrences per horse per test).

### Heart Rate

Horse heart rate was recorded using Polar Equine H10 sensor (RR recordings; Polar Electro OY, Kempele, Finland), consisting of a belt/elastic girth with two electrodes, a wireless heart rate sensor and a wristwatch receiver. The belt with electrodes was fitted around the horse’s belly behind the shoulder. Water and ultrasound gel [Apotekets Eksplorations gel (clinic), 211894, Skovlunde, Denmark] were applied for optimal connection between electrode and skin. Data were downloaded using the online software Polar Flow. Average and maximum heart rate from habituation, target training, choice test, visible displacement task, and invisible displacement task, respectively, were noted. Heart rate variability was not considered as the duration of each trial or registration period (either habituation, training or testing period) was shorter than 10 min each (von Borell et al., 2007).

### Data Handling and Editing

All trials were video recorded and coded live while testing. All choices of the horses (left or right side and corresponding correct or incorrect) were clear and unambiguous; hence, we did not calculate inter-observer reliability.

One horse showed strong neophobic reactions during the habituation period and was thus excluded from the study for welfare reasons. All remaining horses complied with the habituation criterion (both step 1 and step 2) and all but one horse complied with the target training success criterion and thus proceeded to the choice test (i.e., data from habituation *n* = 39, and target training *n* = 38). From the choice test, all but one horse (which succeeded only in 50% of trials) complied with the success criterion (Table 2) and hence 37 horses proceeded to the visible displacement task and the invisible displacement task. During the visible displacement task, two horses lost interest in the test (did not approach the board/the task upon presentation), resulting in 35 horses in the analysis of the visible displacement task. In the invisible displacement task, the same two horses did not show an interest upon presentation of the task, and four other horses dropped out after the first five (unsuccessful) trials and hence were excluded from the analysis of the invisible displacement task (Table 2). In addition, there were some errors in the heart rate measures, potentially due to low conductance between skin and electrode when horses moved. This resulted in the following sample sizes for the heart rate data for the different phases of the experiment (Table 2).

**TABLE 2:** Overview of the sample sizes from the different parts of the experiment: Target training, choice test, visible displacement task, invisible displacement task, and sub-sample sizes from the heart rate equipment data.

Part of experiment (heart rate = HR)	Sample size (n)
Target training	38
- Target training, average HR	38
- Target training, maximum HR	37
Choice test	37
- Choice test, average HR	33
- Choice test, maximum HR	32
Visible displacement task	35
- Visible displacement task, average HR	33
- Visible displacement task, maximum HR	33
Invisible displacement task	31
- Invisible displacement task, average HR	28
- Invisible displacement task, maximum HR	29

### Statistics

Statistical analyses were performed using the R software, version 3.1.2 (R Core Team, 2019) and all *p*-values evaluated according to a significance level of 5%.

#### Performance

To investigate if individual horses performed better than chance (50% correct choices out of 10 trials), binomial tests were used. To test the group performance in the tests, one-sample Wilcoxon tests with chance level set at 50% were used. To analyze which individual characteristics affected performance of the horses, a Generalized Linear Mixed-effects model was fitted to the data from each test (i.e., three models). Each full model included: training level (categorical variable with three levels: T0–2, T3, T4–5), age (numeric variable, mean ± SD = 12 ± 7.5), sex (categorical variable with two levels: male/female), color (categorical variable with two levels: red, not red), motivation to the task (continuous variable; trials per minute during target training, mean ± SD = 3.7 ± 1.8), baited side (categorical variable with two levels: left/right), and for the visible and invisible displacement tasks the displacement direction in each trial (categorical variable with two levels: toward/away from the horse) on performance in the tests. As the primary response variable was binary (i.e., successful or not according to the outcome from the binary tests i.e., 1/0), data was analyzed using a logistic regression with package lme4 (Bates et al., 2015) in the R software. The full models included horse ID as the random term to account for repeated measures on each horse (i.e., 10 trials per horse), all explanatory variables as listed above as main fixed effects, and interaction between age and training level. If the full models failed to converge (full model for the visible and invisible displacement task), an optimizer (“bobyqa”) was included. Model assumptions were checked using the package DHARMa (Hartig, 2021).

Spearman correlation analyses were lastly carried out to test if successful horses in the visible displacement task also were more successful in the invisible displacement task (correlations between choice test and the other tests were not done due to the high success rate, and thus low variability in responses, in the choice test).

#### Heart Rate

In the analyses of heart rate data, Linear Mixed-effects models were fitted for mean heart rates and maximum heart rates, respectively, using packages lme4 (Bates et al., 2015) and nlme (Pinheiro et al., 2019). Mean performance of the horses were included as response variable, and Horse ID was again included as the random term to account for repeated measures on each horse. Test (choice, visible displacement task, invisible displacement task) and heart rate (either mean or maximum) was included as fixed effects in the full models. Pearson correlation analyses were also conducted to analyze if horses with high heart rate in one test also had a high heart rate in the other tests.

#### Behavior

Linear Mixed-effects models were used to elucidate if behavior indicative of frustration was linked to successful or unsuccessful trials, and to assess if an association between success rate and incidence of the behavior occurred, using the same model as mentioned above for heart rate analysis, with the number of frustration behaviors being fitted in the model.

## Results

### Performance

**FIGURE 3:**
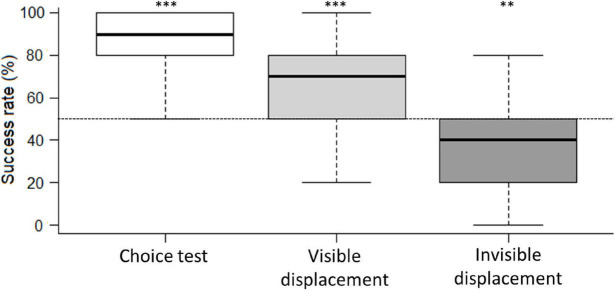
Boxplot of the group’s success rate (%) in choice test, visible displacement task and invisible displacement task. The boxes represent the 25; 75% quartiles with the black line representing the median. The dashed vertical bars represent the range (i.e., minimum and maximum) within the group. The chance level (50%) is marked by the horizontal dashed line. ***P < 0.001 and **P < 0.01 in the one-sample Wilcoxon test, illustrates that the horses as a group performs better/worse than expected by chance in the choice test and the visible displacement task, but worse than expected by chance in the invisible displacement task.

Individual horses needed to complete 9/10 correct trials in order to perform significantly better than expected by chance (binomial test based on 10 trials, *p* < 0.05). In the first 10 trials of the choice test, 27 horses performed better than expected by chance, and eight horses performed better than expected by chance in the visible displacement task. In the invisible displacement task, none of the horses performed significantly better than expected by chance (Table 3). This picture was also reflected in the group performance where horses performed above chance level in choice test and visible displacement task (One-sample Wilcoxon test, *p* < 0.05, Figure 3). In the invisible displacement task, the group performed significantly below than expected by chance (one-sample Wilcoxon test, *p* < 0.05, Figure 3) indicating that horses persistently chose the side where they had last seen the target (i.e., where they had seen the target before it was moved while occluded).

**TABLE 3:** Overview of individual performance in choice test, visible displacement task, and invisible displacement task.

Horse ID	Age	Sex	Coat color	Training level	Choice test success	Visible displacement task success	Invisible displacement task success
1	22	F	Dark brown	T4	**10**	8	5
2	5	F	Black	T4	8	6	4
3	4	F	Dark brown	T1	**10**	5	3
4	4	F	Brown	T1	**9**	**9**	5
5	20	M	Palomino	T4	**10**	8	5
6	5	M	Black	T2	**10**	6	3
7	17	F	Red	T4	**9**	**9**	5
8	3	F	Red	T0	8	7	6
9	3	F	White and red	T1	**9**	NA	NA
10	3	F	Red	T0	NA	NA	NA
11	17	F	Black	T3	**10**	4	1
12	20	F	Black and white	T3	8	8	2
13	24	F	Brown	T3	**9**	8	2
14	2	F	Gray and white	T0	8	4	2
15	17	F	Black	T3	**9**	5	4
16	24	F	White	T3	**10**	**10**	8
17	20	F	Red	T3	**10**	8	3
18	10	F	Yellow	T4	5	4	NA
19	4	F	Black	T1	NA	NA	NA
20	10	F	Black	T3	**9**	6	1
21	15	F	Yellow	T3	8	8	4
22	20	M	Brown and gray	T3	**10**	**9**	6
23	25	M	Gray	T3	**10**	8	4
24	4	M	Gray	T4	8	3	5
25	7	F	Black	T5	8	2	1
26	10	F	Red	T4	**9**	**9**	7
27	18	F	Red	T3	8	4	NA
28	8	F	Red	T4	**9**	5	4
29	13	F	Red	T3	**9**	3	NA
30	20	F	Red “vindott”	T3	8	5	NA
31	4	M	Black	T3	**9**	4	2
32	4	M	Dark brown	T3	**10**	5	0
33	6	F	Black and white	T5	**10**	8	8
34	8	F	Red	T5	**10**	**9**	0
35	5	F	Black	T3	**10**	**10**	7
36	7	F	Yellow	T5	**10**	6	0
37	5	F	Black	T2	**9**	NA	NA
38	4	F	Black	T2	**9**	8	3
39	4	F	Black	T2	**9**	**9**	2

*Individuals performances significantly higher than chance level (50% correct choices) are indicated in bold [9 or more correct out of 10 trials; Binomial test (two-sided), p < 0.05].*

The fitting of the three models showed that none of the fixed effects [i.e., interaction (age:Training level), Sex, color, motivation to the task, baited side, and for visible and invisible displacement tasks: displacement direction] had a noteworthy effect on horse performance in any of the three tests (GLMM, all variables *p* > 0.05).

There was a positive correlation between success rate in the visible and invisible displacement task, meaning that successful horses in the visible displacement task also were more successful in the invisible displacement task (figure can be found under Supplementary Figure 1).

### Heart Rate

**FIGURE 4:**
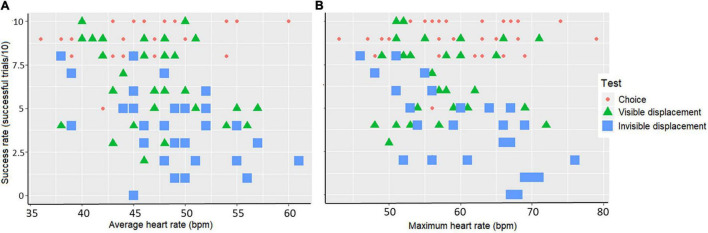
Scatterplot of the association between success rate (successful trials/10) per horse and **A**) average heart rate (bpm) or **B**) maximum heart rate (bpm) during the three tests: choice test (red dots), visible displacement task (green triangles), and invisible displacement task (blue squares).

Overall horses performing well had lower mean and maximum heart rate compared to less successful horses, with the invisible displacement task provoking the highest heart rates and lowest success rates. The model fitting showed that performance were significantly associated with average (*F*_df_ = 83.360_60_ and *p* < 0.001) and maximum (*F*_df_ = 79.558_58_ and *p* < 0.001) heart rate during the tests. The model output moreover showed that low average and low maximum heart rate were significantly associated with high performance in the invisible displacement task [Linear Mixed-effects model: (mean) *F*_df_ = 9.06_1_, *p* < 0.001 and (max) *F*_df_ = 22.28_1_ *p* < 0.001] but not in the visible displacement task [Linear Mixed-effects model: (mean) *F*_df_ = 1.84_1_, *p* = 0.18 and (max) *F*_df_ = 0.25_1_, *p* = 0.62] and the choice test [Linear Mixed-effects model: (mean) *F*_df_ = 1.98_1_, *p* = 0.28 and (max) *F*_df_ = 0.47_1_, *p* = 0.45] (Figures 4A,B).

The Pearson correlations showed that horses with high (or low) heart rates in one test also had high (or low) heart rates in the next test (figure can be found under Supplementary Figure 2).

### Behavior

The occurrence of behavior indicative of frustration was generally low, but increased from Choice test to the visible displacement task and again from the visible displacement task to the invisible displacement task. In the choice test, behavior indicative of frustration was noted in 6% of the trials (22 out of 370 trials), and of these, the majority of trials were unsuccessful [5 out of 22 trials were successful, Linear Mixed-effects model: *F*_df_ = 52.81_1_, *p* < 0.001]. In the visible displacement task, horses expressed behavior indicative of frustration in 23% (82 of 350 trials) of the trials, and in the invisible displacement task, the percentage further increased to 43% (134 of 310 trials), but the behavior was still mainly linked to unsuccessful trials [Linear Mixed-effects models: (visible displacement task) *F*_df_ = 82.79_1_, *p* < 0.001 and (invisible displacement task) *F*_df_ = 55.18_1_, *p* < 0.001].

## Discussion

The current study tested horses’ performance in visible and invisible displacement tasks and investigated whether specific ontogenetic, behavioral and physiological factors were associated with performance. Horses performed above chance on group and individual level in the visible displacement task, but not the invisible displacement task. In the invisible displacement task, the group performed significantly below than expected by chance, indicating that horses persistently chose the side where they had last seen the target before it was occluded and moved. None of the individual characteristics included in the study (age, sex, training level, and coat color) had an effect on performance of the horses. Overall, horses performing well in the displacement tasks had lower mean and maximum heart rates, and this association was most pronounced in the invisible displacement task, where unsuccessful horses had the highest mean and maximum heart rates measured during the experiment. Behavior indicative of frustration was linked to the amount of incorrect trials across all tests. These differences in behavioral and physiological parameters, as well as their potential interplay with performance in the tasks, are relevant to make adequate comparisons between individual test performances and should be the focus of future research.

The finding that horses were able to solve the visible displacement task but not the invisible one was in accordance with our hypothesis and with previous findings by Trösch et al. (2020). In our study, however, the results were achieved using a larger sample (>30 horses) compared to the study by Trösch et al. (2020) (<20 horses). In contrast to other studies of object permanence, the target in our study was not moved behind a barrier, but was instead covered by a white cup. In the visible displacement task, this could have allowed the horses a slightly longer latency to look at the target in the right location before it was shielded, and hence could have influenced the success rate positively. However, comparing the high number of individual successful horses in the choice test (27 out of 37) to the visible displacement task (8 out of 35), the strong difference indicates that even when visible, not all horses are able to follow an object—or show a perseveration error to the location where the object was first seen. On the other hand, the high number of successful horses in the choice task indicates that horses are good at remembering the location of objects. It also aligns with previous findings that horses are able to remember the location of hidden food (McLean, 2004; Baragli et al., 2011) and also companions disappearing behind a barrier (Proops et al., 2009). On the other hand, the ability of following objects even when not visible might have little biological relevance to horse, and hence might not have been favored during evolution. In forests, this ability could have been favored as it could aid predator vigilance (i.e., the ability to spot and follow predators even when hidden). However, in open areas, such as grasslands and meadows, which horses evolved in, predators are rarely hidden as trees or bushes are absent, making the need for tracking the movement of hidden objects less relevant.

The current study found a correlation between performance in the visible and invisible displacement task which was expected and implied that some horses were generally more successful than others. In addition to this, heart rates were also positively correlated between tests, implying that some horses generally had higher/lower heart rates. Collectively, these findings point to stable inter-individual differences, which should be the focus for future studies. We moreover hypothesized that training level would influence the performance of the horses positively, as trained horses were expected to pay more attention to the human experimenter and/or be less fearful toward the experimenter and the task. The lack of effect of training level on performance in the tests was thus surprising. This lack of effect from prior training could be explained by the nature of these particular tasks, which arguably was very different from what horses were usually exposed to during training (i.e., riding, longing or round pen training). The lack of effect of any of the other individual characteristics (coat color, age, sex) was also unexpected. Hence, these findings contrast previous findings from other cognitive tasks where younger horses performed better than older horses (Murphy et al., 2004; Krueger et al., 2014; Kjellberg and Morgan, 2021), and studies where sex differences were found (Wolff and Hausberger, 1996; Murphy et al., 2004). In the latter case, the results needs to be viewed in the context of the slightly skewed sample size with 7 males and 31 females included. This also yields a skewed distribution of successful horses in the visible displacement task, with only 1 out of the 8 successful horses being male, which warrants further investigation of this particular factor. On the other hand, the underlying reason for the contrasting results might again be due to the nature of this particular task. The task has both instrumental and visuo-spatial components, and as female horses are better instrumental problem solvers (Wolff and Hausberger, 1996), while males outperform females in visuo-spatial tasks (Murphy et al., 2004), the combination of both aspects might have offered the sexes equal opportunities of solving the task. In terms of coat color, we hypothesized red or chestnut colored horses to be less successful in the tasks, due to the common anecdotal assumption that these horses have a general higher sensitivity to tactile stimuli (e.g., Rørvang et al., 2020a). Although it may be fair to argue that higher tactile sensitivity might affect performance in other cognitive tasks (e.g., learning to avoid pressure i.e., a tactile stimulus), this was not confirmed for this test on object permanence. In order to further the research on the effects of individual characteristics, future studies might focus on additional traits such as e.g., curiosity/exploratory behavior (as argued by Christensen et al., 2021b), shyness/boldness, dominant/submissive (as argued by Lansade and Simon, 2010) both in terms of willingness to engaging in new tasks but also in terms of approaching a human, as potential sources of variation in cognitive tasks.

The results showed that horses expressed both behavioral and physiological signs of frustration when they were not able to solve the presented displacement task. This finding is in line with previous studies where horses were faced with different tasks [e.g., when trying to get hay from triple tier hay nests (Ellis et al., 2015), solving an operant task: (Rørvang et al., 2020b), and during locomotion (Ödberg, 1973)] and also expressed behavior indicative of frustration. Adding to this, the behavioral frustration data was obtained by 1/0 sampling which means the true frequency of frustration behavior is likely much higher. Although we were not able to disentangle cause and effect with our setup, the increased frustration/arousal could have led to a negative feedback loop that might hamper subject’s performance in subsequent trials. Future tests should aim to control for arousal levels in their design, and might also include baseline measurements of arousal, in order to decrease individual variation and to safeguard comparability of results.

### Practical Implications

Horses’ ability to solve visible displacements and their ability to remember placement of hidden food is beneficial as it may aid their ability to follow objects, conspecifics and humans in their environment. On the other hand, horses’ inability to follow hidden objects is also important information as it means that one should not expect a horse to know where objects, conspecifics or humans have gone, if the move or displacement was not visible to the individual. As a result, horses may react fearful toward suddenly re-appearing objects, conspecifics or humans, which could be unexpected by humans, which poses a risk of mismatching expectations. Therefore, it is important to know and communicate both the abilities but certainly also the inabilities of horses to ensure welfare and a safe horse training environment for both horses and humans. The frustration shown in both the occurrence of conflict behavior and heart rate associated with non-successful trials further pinpoints this issue.

In terms of practical horse training, horse’s ability to follow and remember the placement of targets is beneficial in situations where the trainer would want to distance him/herself from the horse. This could for instance be in risky situations such as during training for loading to transport. In such close contact situations, horses may react fearfully and unexpected (due to the novelty of the situation), and hence using targets on e.g., a stick, could be a way to train the horse from a safe distance (see e.g., Dai et al., 2019).

## Conclusion

In this study, horses were able to solve a visible displacement task, but were not able to follow invisible displacements. None of the individual characteristics [neither training level, age, sex, nor coat color (red/other colors)] affected performance of the horses. Horses performing well in the displacement tasks had lower mean and maximum heart rates, and showed lower levels of frustration behavior. In practical terms, we can expect horses to follow and remember the previous placement of visible objects in their environment, but we cannot expect horses to follow these objects if moved while occluded from the horses. The results also show that horses express both behavioral and physiological signs of frustration when being unable to solve a task. This highlights the need for adaptation of the tasks we ask of horses to ensure they are able to solve them in order to safeguard their welfare.

## Data Availability Statement

The two datasets from this study can be found in Supplementary Data S7, S8. Any further enquiries regarding data should be addressed to MR, mariav.rorvang@slu.se.

## Ethics Statement

The Board for Animals in Research and Teaching at SLU, Sweden reviewed and approved the animal study and all procedures were conducted in accordance with national legislation on animal experimentation by the Danish Ministry of Justice, Act. no. nr. 253 (8 March 2013) and §12 in Act. no. 1459 (17 December 2013), as well as met the ARRIVE guidelines and the ethical guidelines proposed by the Ethical Committee of the ISAE (International Society of Applied Ethology). Written informed consent was obtained from the owners for the participation of their animals in this study.

## Author Contributions

MR initiated the idea for this study, applied for (and was later awarded) funding for the project, was in charge of data management and prepared data for analyses, and conducted the study in virtual collaboration with all authors (due to COVID-19 restrictions). MR and CN designed the experimental protocol adapted to horses. Data and corresponding analyses were discussed by all authors, and MR subsequently performed the statistical analyses in collaboration with CN. KN and MR wrote the first draft of the manuscript. All authors contributed in the discussions of the protocol in the beginning of the experiment and contributed in writing, proofreading, and fine-tuning the manuscript for publication.

## Conflict of Interest

The authors declare that the research was conducted in the absence of any commercial or financial relationships that could be construed as a potential conflict of interest.

## Publisher’s Note

All claims expressed in this article are solely those of the authors and do not necessarily represent those of their affiliated organizations, or those of the publisher, the editors and the reviewers. Any product that may be evaluated in this article, or claim that may be made by its manufacturer, is not guaranteed or endorsed by the publisher.

## Supplementary Material

Supplementary Figure 1 Positive correlation between success rate in the visible displacement task and the invisible displacement task. The black line represents the regression line with the gray area representing the confidence interval. Correlation coefficient and *p*-value for the test is given in the plot.

Supplementary Figure 2 Pearson correlation analyses between **(A)** average heart rate during choice test with average heart rate during the visible displacement task, **(B)** maximum heart rate during choice test with maximum heart rate during the visible displacement task, **(C)** average heart rate during the visible displacement task with average heart rate during the invisible displacement task, and **(D)** maximum heart rate during the visible displacement task with average heart rate during the invisible displacement task. Correlation coefficients and *p*-values from the tests are given for each correlation.

Supplementary Video 1 Video illustrating the target training.

Supplementary Video 2 Video illustrating a successful horse in the visible displacement task.

Supplementary Video 3 Video illustrating a successful horse in the invisible displacement task.

Supplementary Data 1 Data from the study summarized by horse (i.e., one line per horse).

Supplementary Data 2 Data from the study by trials (i.e., each line represents one trial per horse).
