# Supplementary figures and images for: Horses’ (Equus caballus) Ability to Solve Visible but Not Invisible Displacement Tasks Is Associated With Frustration Behavior and Heart Rate
Source: Front Behav Neurosci. 2021 Dec 8;15:792035. doi: 10.3389/fnbeh.2021.792035 (PMC8693624; doi:10.3389/fnbeh.2021.792035)

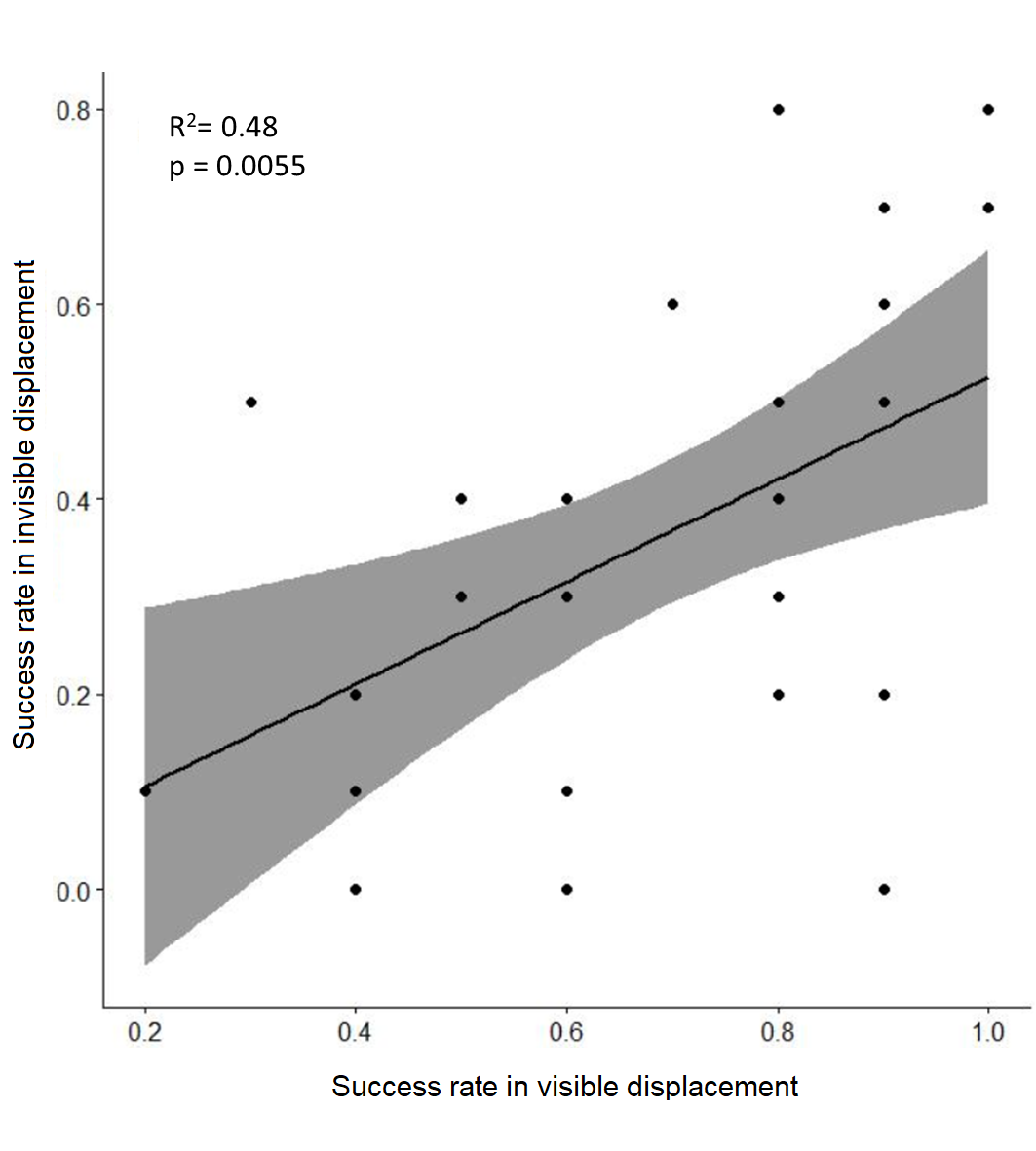

Supplement: Supplementary Figure 1 — Positive correlation between success rate in the visible displacement task and the invisible displacement task. The black line represents the regression line with the gray area representing the confidence interval. Correlation coefficient and p-value for the test is given in the plot. [file Image_1.tif]

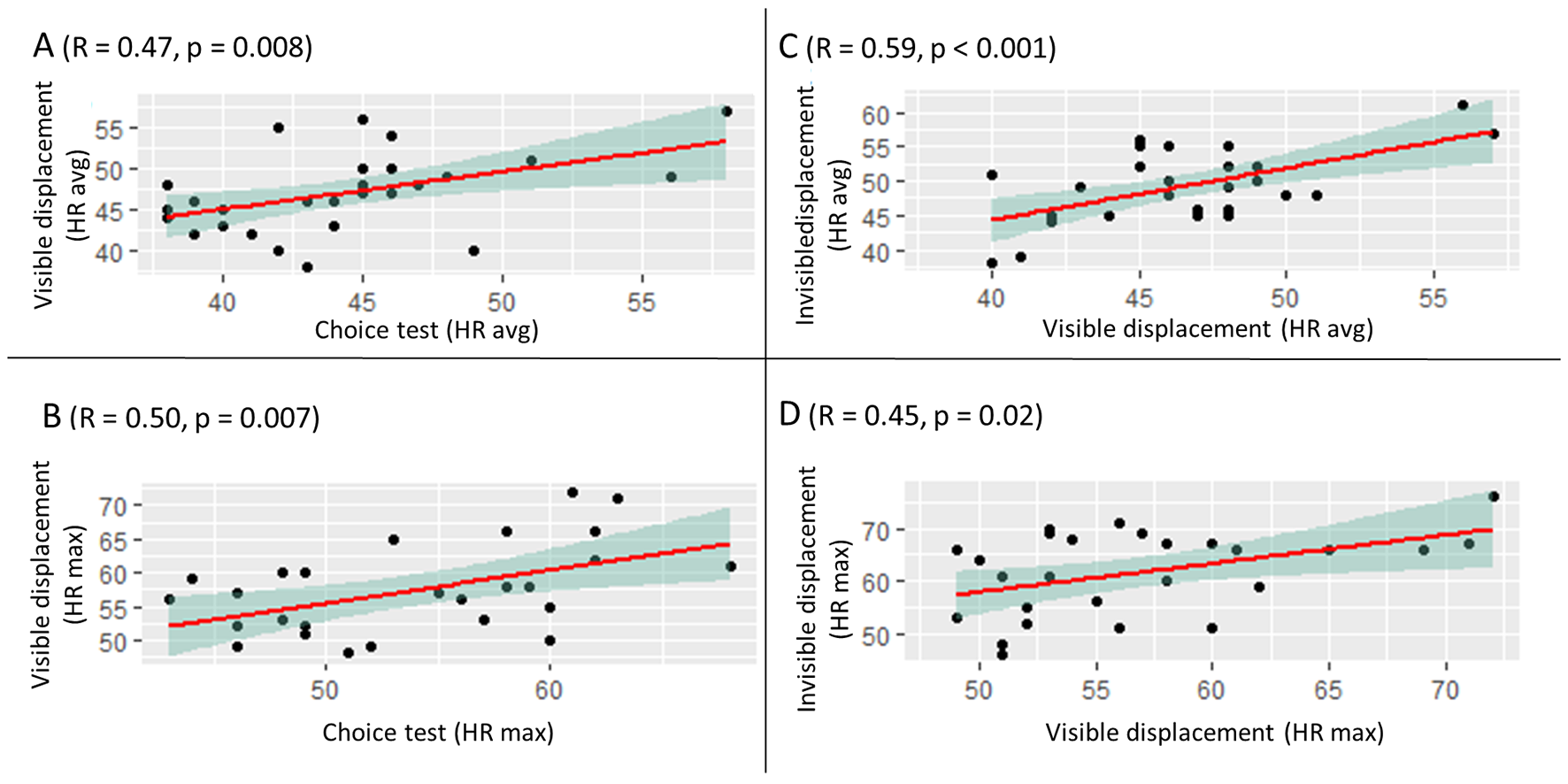

Supplement: Supplementary Figure 2 — Pearson correlation analyses between (A) average heart rate during choice test with average heart rate during the visible displacement task, (B) maximum heart rate during choice test with maximum heart rate during the visible displacement task, (C) average heart rate during the visible displacement task with average heart rate during the invisible displacement task, and (D) maximum heart rate during the visible displacement task with average heart rate during the invisible displacement task. Correlation coefficients and p-values from the tests are given for each correlation. [file Image_2.tif]
